# Contribution of portable obstetric ultrasound service innovation in averting maternal and neonatal morbidities and mortalities at semi-urban health centers of Ethiopia: a retrospective facility-based study

**DOI:** 10.1186/s12884-022-04703-1

**Published:** 2022-04-28

**Authors:** Hailemariam Segni Abawollo, Zergu Tafesse Tsegaye, Binyam Fekadu Desta, Ismael Ali Beshir, Birhan Tenaw Mengesha, Asfaw Adugna Guteta, Atrie Fekadu Heyi, Tsega Teferi Mamo, Zenawork Kassa Gebremedhin, Heran Demissie Damte, Meseret Zelealem, Mesele Damte Argaw

**Affiliations:** 1 USAID Transform: Primary Health Care Activity, JSI Research & Training Institute Inc, Addis Ababa, Ethiopia; 2 Ministry of Health, Maternal and Child Health Directorate, Addis Ababa, Ethiopia

**Keywords:** Obstetric ultrasound, Averted maternal and neonatal morbidities and mortalities, Averted maternal and neonatal deaths, Averted morbidities and mortalities, USAID Transform: PHC, Ethiopia

## Abstract

**Background:**

The maternal and neonatal mortalities in Ethiopia are high. To achieve the Sustainable Development Goals, innovations in ultrasound scanning and surveillance activities have been implemented at health centers for over 2 years. This study aims to estimate the contribution of obstetric ultrasound services on averted maternal and neonatal morbidities and mortalities in Ethiopia.

**Methods:**

A retrospective facility-based cross-sectional study design was conducted in 25 selected health centers. Data were extracted from prenatal ultrasound registers. SPSS version 25 was used for analysis. To claim statistically significant relationship among sartorial variables, a chi-square test was analyzed and *P* < 0.05 was the cut-off point.

**Results:**

Over the 2 years, 12,975 pregnant women were scanned and 52.8% of them were residing in rural areas. Abnormal ultrasound was reported in 12.7% and 98.4% of them were referred for confirmation of diagnosis and treatment. The ultrasound service has contributed to the prevention of 1,970 maternal and 19.05 neonatal morbidities and mortalities per 100,000 and 1,000 live births respectively. The averted morbidities and mortalities showed a statistically significant difference among women residing in rural and semi-urban areas, X,^2^ df (10) = 24.07, *P* = 0. 007 and X,^2^ df (5) = 20.87. *P* = 0.00, 1 respectively.

**Conclusion:**

After availing the appropriate ultrasound machines with essential supplies and capacitating mid-level providers, significant number of high-risk pregnant women were identified on time and managed or referred to health facilities with safe delivery services. Therefore, scaling-up limited obstetric ultrasound services in similar setups will contribute to achieving the Sustainable Development Goals by 2030. It is recommended to enhance community awareness for improved utilization of ultrasound services by pregnant women before the 24th week of gestational age.

**Supplementary Information:**

The online version contains supplementary material available at 10.1186/s12884-022-04703-1.

## Background

In the year 2017, an estimated 295,000 women died globally during and following pregnancy and childbirth. Low-resource settings are where almost all of these deaths (94%) occurred. The problem is considerably worse in the Sub-Sharan Africa (SSA) region, from where two-thirds of the maternal deaths were reported [1]. The following year (2018), the World Health Organization (WHO) estimated a worldwide death of 2.5 million neonates [2]. Another 2.6 million babies were stillborn. Almost all (99%) of these adverse birth outcomes occurred in low- and lower-middle-income countries where half of the deaths happened at home [3].

More than half (52%) of pregnant women give birth outside of health facilities in Ethiopia. In 2017, the estimated maternal mortality ratio and neonatal mortality rate in Ethiopia were 401 per 100,000 live births and 27.5 per 1000 live births, respectively [4]. The direct causes of maternal deaths in Ethiopia are documented to be severe bleeding mainly after childbirth (29.9%), obstructed labor or ruptured uterus (22.3%), pregnancy related high blood pressure (16.9%), puerperal sepsis (14.6%), and unsafe abortions (8.6%) [5]. Moreover, in line with the WHO fact sheet (2020), in Jimma Referral Hospital, south-western Ethiopia similar causes of neonatal admissions and deaths in the intensive care units were identified [6]. The top causes of neonatal deaths were low birth weight, prematurity, respiratory distress syndrome, sepsis, hypoglycemia, congenital malformations, and pathologic jaundice [6, 7].

All women have the right to have access to high quality care during pregnancy, childbirth, and after childbirth [8]. Cognizant of this, the WHO (2016) recommended at least one routine ultrasound (U/S) scanning service before 24th week of gestation as a component of positive pregnancy experience. In addition, to get a positive perinatal health outcome, offering quality antenatal care (ANC) and safe delivery services are also recommendations [9, 10]. A major role of U/S is the accurate confirmation of gestational age which is critical in settings where women often tend to not remember their exact conception dates. It also helps to reduce the number of unnecessary interventions. U/S could also play a major role in reducing adverse maternal outcomes, mainly “near miss” morbidity and mortality. Maternal conditions directly contribute to perinatal outcomes and up to 37% of patients are potentially misdiagnosed. This could be corrected by incorporating U/S services in their care. U/S services may also result in the recognition of conditions that could otherwise have been missed and resulted in adverse outcomes such as placenta previa, adherent placenta, undiagnosed multiple pregnancies, and malpresentations—leading to life-saving interventions in up to half of pregnant women [11].

Portable point of care U/S technology is being used in several low-income countries across the world [12–23]. More specifically, there is empirical evidence on the relationship between use of limited obstetric U/S scanning services and improved quality of ANC services [17–22]. The obstetric U/S avoids three delays, i.e., in seeking care, in reaching lifesaving care and in care delivery [12, 13, 19, 20, 23]. Nonetheless, there is limited evidence on the contribution of the limited obstetric U/S service innovation in preventing maternal and neonatal deaths in Ethiopia. Therefore, the aims of this study are to describe the U/S service beneficiaries and to investigate the innovation’s contribution towards efforts of averting maternal and neonatal morbidities and mortalities.

## Methods

### Study setting and population

Ethiopia has adopted a federal government structure by establishing 11 regional states and two city administrations [24]. More than 80% of the population lives in rural areas. The national health system is divided into three tiers. Primary healthcare is led by a health center and typically five satellite health posts, targeting 25,000 people [24]. A health center is expected to provide health promotion, disease prevention, curative, and rehabilitative outpatient care including basic laboratory and pharmacy services with a capacity of 10 beds for emergency and delivery services [25]. This study targeted Amhara, Oromia and Southern Nations Nationalities and Peoples’ (SNNP) regions of the country. These regions were purposively selected with a criterion where limited obstetric U/S scanning services were introduced at the health center level to improve quality and equity of prenatal care for over 2 years. There are about 82.6 million residents in the study targeted regions. In line with the long-term outcomes of the United States Agency for International development (USAID) Transform: Primary Health Care project, 100 health centers were selected and supported with portable U/S machines [26].

### Study design and period

A retrospective facility-based cross-sectional study design was employed to estimate the contribution of introduction of obstetric U/S services at health centers on averted maternal and neonatal morbidities and mortalities between January 2019 and December 2020.

### Intervention

In Ethiopia, to increase access to healthcare technology among pregnant women of rural residents, the USAID Transform: Primary Health Care project introduced innovative U/S scanning services at the health center level, which are located close to rural communities of Ethiopia [26, 27]. To initiate the services, in October 2018, the project capacitated mid-level health professionals (mainly midwives) to offer limited obstetric U/S scanning services through task shifting/sharing principles [28, 29]. The knowledge and skill building activity was executed through a 10-day classroom basic limited obstetric ultrasound training supplemented with experiential learning events under supervision of Gynecologist/Obstetricians and Radiologists. In addition, three sessions of objective structured clinical examination (OSCE) followed by onsite and offsite mentoring/coaching sessions were facilitated [30]. A competent certified mid-level health professional can operate ultrasound machines and identify normal pregnancy, first trimester pregnancy and complications, fetal dating and measurements, second and third trimester pregnancy and complications. In addition, the trained and competent health professionals were equipped with the necessary equipment, supplies, and a place to refer women to. This was intended to enable health professionals to confirm pregnancies using U/S scanning services and identify high risk cases, to link women with the next level health facility that has functional Emergency Obstetric and Newborn Care (EmONC) services—ultimately ensuring safe deliveries [26, 27].

### Sample size and sampling

**Fig. 1:**
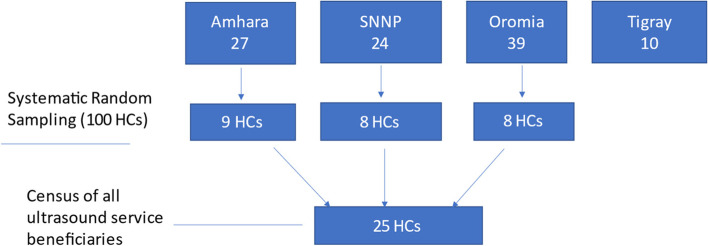
Schematic presentation of sampling techniques, 2020

The sample size was determined based on the recommended rule of thumb that if the health centers are between 50 and 100, a 20 to 30% sample should be taken [31]. Hence, out of the 100 health centers providing limited obstetric U/S scanning services, for this study, the investigators sampled 25 health centers and selected targeted facilities using systematic random sampling techniques, where the sampling interval (k) is 3 (Fig. 1). Once the health center was identified, the information of all prenatal obstetric U/S service beneficiaries was included in the study.

### Data collection and management

The data collectors were 25 midwives who attended basic U/S scanning training and were also trained for 2 days on the tools and principles of data collection. In addition, five supervisors who are experts on maternal and neonatal health services were assigned to check and maintain the quality of collected data.

As part of introducing U/S services in Ethiopian health centers, a logbook was developed and distributed to each intervention health facility (Additional file 1). The questionnaire was developed after reviewing relevant literatures [12, 13, 15–17, 20, 29]. The data sheet consists of demographic information of obstetric U/S service beneficiaries, including medical record number, age, and residential address. In addition, the trained mid-level health professionals who operated the U/S machines documented the indications for U/S scanning, gestational age estimated based on Last Menstrual Period (LMP) and U/S scanning findings, fetal biometry measurements, U/S diagnosis, action taken, and reasons for offered referral services. This information was extracted and entered to a Microsoft Excel 2016 (Microsoft Inc., Seattle WA) spreadsheet program for data storing, transferring, and cleaning.

### Averted morbidities and mortalities

Using a predefined criterion, the abnormal U/S scanning reports were categorized based on their potential to cause catastrophic maternal and neonatal health outcomes. The magnitude of risk for maternal morbidities and mortalities were estimated as percent of possible maternal morbidities and mortalities over total ultrasound scan through considering cases of fetal malpresentation, abnormal placentation, multiple fetus, small or large gestation age, intrauterine fetal death (IUFD) or fetal demise, gross anomalies, abnormal fluids, abortion, ectopic pregnancy, and pelvic pathology as a numerator, and all U/S scanning report as denominator. In addition, the possibility of neonatal morbidity and mortality was estimated using percent of possible neonatal morbidities and mortalities over total ultrasound scan through the following cases of abnormal U/S scanning reports: fetal malpresentation, oligo-hydramnios, polyhydramnios, small or large for gestation age, and multiple fetus as a numerator, and all ultrasound scanning reports were the denominator. Therefore, the investigators decided to consider all possible risks for maternal and neonatal morbidities and mortalities in risk averted estimations, which can be reduced through confirmation using advanced perinatal health services accessed through referral linkage.

The dependent variables were abnormal U/S scanning surveillance reports with possibilities of catastrophic maternal and neonatal perinatal health outcomes.

The independent variables were residential addresses and intervention regions.

The inclusion criteria were all Vscan limited obstetric ultrasound service beneficiaries during ANC from January 2019 through December 2020.

The exclusion criteria were ANC service beneficiaries between January 2019 and December 2020 who did not receive ultrasound scanning services.

### Data analysis

First, descriptive statistics for all dependent and independent variables were calculated. The age of pregnant women, a continuous variable, was summarized using mean [± standard deviation (SD)]. The rest of the categorical variables were presented using frequencies and percentages. The Pearson Chi-square test was used to evaluate the differences among categorical variables. The statistical differences were claimed at *P*-value < 0.05. IBM SPSS Statistics for Windows, version 25 was used in the data analysis [32].

## Results

### Characteristics of the study population

The average distance of a referral receiving hospital from health centers with obstetric U/S services is 53.6 km with the longest distance found in the Oromia Region (Table 1).

**Table 1:** Characteristics of the study population, USAID Transform: Primary Health Care intervention areas, 2019-2020

Characteristics	Amhara	Oromia	SNNP	Total
Number of health centers	9	8	8	25
Average distance from referral receiving facility in kilometers	37.3	87.1	38.4	53.6
Population				
Catchment population	330,083	329,222	242,815	902,120
Eligible pregnant women				
2019	12,106	19,464	8936	40,506
2020	12,421	19,970	9168	41,559

### Antenatal ultrasound scanning services

A total of 12,975 pregnant women were scanned at the 25 health centers over the two-years period (2019-2020). Slightly higher than half (52.8%) of the scanned women were residing in rural areas and were with an overall mean age of 27.7 (±5.2) years. Only around a third (31.8%) of the women recalled the exact date of their conception. Despite the need for an early U/S scanning in such settings where most women do not know their date of conception, only 5.2% of the scanned women were scanned during their first trimester of pregnancy. Almost all (92.2%) of the women were scanned as part of routine ANC services. Of the total scanned women, 12.7% of them were found to have abnormal U/S reports, and almost all (98.4%) of those with abnormal reports were referred to a nearby hospital for confirmation of diagnosis and subsequent treatment (Table 2).

**Table 2:** Antenatal U/S scanning service data of pregnant women by regions, USAID Transform; Primary Health Care intervention areas, 2019- 2020

Characteristics	Amhara	Oromia	SNNP	Total
Ultrasound scanning	4957(38.3%)	5222(40.2%)	2796(21.5%)	12,975(100%)
Age of mothers (mean ± SD) in years	27.8 **±** 5.3	26.8 **±** 4.5	26.4 **±** 4.4	27.7 **±** 5.2
Residential address				
Semi-urban	2406(48.5%)	2619 (50.2%)	1105 (39.5%)	6130 (47.2%)
Rural	2551(51.5%)	2603 (49.8%)	1691 (60.5%)	6845 (52.8%)
Women that know their LMP date	2663 53.7%)	1028 (19.7%)	438 (15.7%)	4129 (31.8%)
Indication for ultrasound scanning services				
Routine services	4522(91.2%)	4828 (92.5%)	2609 (93.3%)	11,959 (92.2%)
High risk pregnancy	421(8.5%)	368 (7.0%)	169 (6.0%)	958 (7.4%)
Emergency	14 (0.3%)	26 (0.5%)	18 (0.6%)	58 (0.4%)
Frequency of ultrasound scanning				
First ultrasound scanning	4440 (89.6%)	4779 (91.5%)	2278 (81.5%)	11,497 (88.6%)
Second ultrasound scanning	396 (8.0%)	310 (5.9%)	269 (9.6%)	975 (7.5%)
Third ultrasound scanning	119 (2.4%)	121 (2.3%)	243 (8.7%)	483 (3.7%)
Time of ultrasound scanning				
First trimester	260 (5.2%)	195 (3.7%)	226 (8.1%)	681(5.2%)
Second trimester	1101 (22.2%)	1387 (26.6%)	1359 (48.6%)	3847(29.6%)
Third trimester	3596 (72.5%)	3640 (69.7%)	1211 (43.3%)	8447(65.1%)
Ultrasound scanning diagnosis				
Normal ultrasound scanning reports	4328 (87.3%)	4580 (87.7%)	2450 (87.6%)	11,358(87.5%)
Abnormal ultrasound scanning reports	629 (12.7%)	642 (12.3%)	346 (12.4%)	1617 (12.7%)
Action taken				
Managed in the facility	16 (2.5%)	7(1.1%)	3 (0.9%)	26 (1.6%)
Referred to next level facility	613 (97.5%)	635 (98.9%)	343(99.1%)	1591 (98.4%)

### Maternal and neonatal morbidities and mortalities averted

The introduction of obstetric U/S scanning services at health centers has contributed to the prevention of 1970 maternal morbidities and mortalities per 100,000 live births at the 25 health centers during the assessed two-years period. Fetal malpresentations of various kinds (mainly breech presentations) were responsible for the referrals to nearby hospitals of 8.9% of the total scanned pregnant women, followed by multiple pregnancies in 1.3% of the cases. The Chi square (X^2^) test of maternal morbidities and mortalities averted through the introduction of U/S services at health centers showed a statistically significant difference among women residing in rural and semi-urban areas, X,^2^ df (10) = 24.07, *P* = 0. 007 (Table 3).

**Table 3:** Averted maternal and neonatal morbidities and mortalities by residence areas of pregnant women, USAID Transform: Primary Health care intervention areas, 2019-2020

Variables	Response category	Residence area						Test statistics	
		Total (*N* = 12,975)		Semi-urban (n_1_ = 6130)		Rural (n_2_ = 6845)			
		Freq.	%	Freq.	%	Freq.	%	X^2^	*p*-value
Ultrasound finding	Normal scans	11,358	87.5	5423	88.5	5935	86.7	9.19	**0.002**
	Abnormal scans	1617	12.5	707	11.5	910	13.3		
Maternal deaths averted	Normal	11,358	87.5	5423	88.5	5935	86.7	24.07	**0.007**
	Fetal malpresentation	1038	8.0	430	7.0	608	8.9		
	Multiple fetuses (twins)	160	1.2	73	1.2	87	1.3		
	Abnormal placentation	131	1.0	55	0.9	76	1.1		
	Small or large for gestational age	98	0.8	54	0.9	44	0.6		
	Abnormal fluid (oligo or polyhydramnios)	80	0.6	43	0.7	37	0.5		
	Gross anomalies	23	0.2	14	0.2	9	0.1		
	IUFD or fetal demise	31	0.2	13	0.2	18	0.3		
	Abortion	29	0.2	12	0.2	17	0.2		
	Ectopic pregnancy	14	0.1	8	0.1	6	0.1		
	Pelvic pathology	13	0.1	5	0.1	8	0.1		
Neonatal deaths averted^a^	Normal scans	11,358	89.0	5423	89.8	5935	88.3	20.87	**0.001**
	Fetal malpresentation	1038	8.1	430	7.1	608	9.0		
	Multiple fetuses (twins)	160	1.3	73	1.2	87	1.3		
	Small or large for gestational age	98	0.8	54	0.9	44	0.7		
	Oligohydramnios	80	06	43	0.7	37	0.6		
	Polyhydramnios	23	0.2	14	0.2	9	0.1		

NB: ^a^218 abnormal ultrasound scanning reports i.e., abortions, ectopic pregnancies and fetal demise were not included in the category of neonatal deaths averted variable; However, the data is doubled for estimated neonatal deaths

Similarly, the introduction of obstetric U/S scanning services at health centers has contributed to the prevention of 19.05 neonatal morbidities and mortalities per 1000 live births at the 25 health centers during the two-year period. The major possible causes of neonatal morbidity and mortality identified through U/S scanning was fetal malpresentation, accounting for 9.0% of the total scanned cases, followed by multiple pregnancies, responsible for 1.3% of the total scanned cases. Pregnant women from rural areas were referred to nearby hospitals to prevent neonatal morbidities and mortalities which was found to be statistically significant at X,^2^ df (5) = 20.87, *P* = 0.001 (Table 3).

## Discussion

Most maternal and neonatal morbidities and mortalities are preventable through the introduction of innovative technologies at both health facility and community levels [33]. An innovation being widely implemented in low-income countries is the enhancement of quality ANC through capacitating mid-level health professionals (mainly midwives) and introducing obstetric U/S scanning services in rural areas for surveillance of maternal and fetal health [34]. In line with WHO recommendations, the Ethiopian Ministry of Health and development partners introduced this tested innovation to prevent maternal and neonatal negative health outcomes during the immediate postpartum period. This facility-based retrospective study was conducted with the aim of describing ultrasound service beneficiaries and investigating averted maternal and neonatal morbidities and mortalities using U/S diagnosis and linking mothers with functional EmONC health facilities. After analyzing obstetric U/S surveillance data for the period of 2019-2020, this study has revealed slightly higher than one-tenth (12.5%) of pregnant women with abnormal U/S scans were identified. Pregnant women were referred to nearby hospitals to prevent the occurrence of possible catastrophic health conditions on themselves and/or their fetuses. In addition, the results of this study demonstrate that a significantly higher proportion of rural residing pregnant women and their fetuses have benefited from the U/S scanning services than their counterpart semi-urban residents. Therefore, the findings of this study can be informative to policy makers, researchers, development partners, and community members on the contribution of the introduction of obstetric U/S scanning services in rural setups, in preventing the morbidities and mortalities of pregnant women and their fetuses.

This study has shown that only one out of six (12,975/82,065) eligible pregnant women were scanned among the residents of targeted health centers. In addition, slightly higher than one-third (34.8%) of antenatal U/S service beneficiaries received the services before completing their second trimester. This finding was much lower than the WHO’s recommendation to offer U/S scanning services for all pregnant women at least once before the 24th week of gestation [9]. This might have occurred as a result of the existing Ethiopian socio-cultural factor of delayed ANC care seeking behavior, by which women seek care upon the sensation of an initial fetal mobility, which mostly occurs around the fifth month of pregnancy (mid-second trimester) [35]. Furthermore, it could be due to three additional factors including firstly, health system related factors of interruption of U/S services due to stockout, turnover of staff, and lack of alternative electric power sources. The second factor could be connected to trained health professionals including busy schedules, staff shortages due to absence from health centers for night assignments, participation in trainings, and annual vacation leave. The third factor could pertain to community related issues such as lack of awareness and fears around U/S machines. These findings were consistent with a study in Kenya that reported on barriers of prenatal U/S service utilization, including lack of money, limited awareness, and fear of side effects [36].

Antenatal U/S scanning services may prevent unexpected and severe health outcomes during the immediate postnatal period. These negative and catastrophic health outcomes may be observed due to unexpected twins, undiagnosed placenta previa, adherent placenta, fetal malpresentations, other maternal health conditions and certain infections (CMV, Rubella), and genetic abnormalities faced during labor and delivery [11]. In this study, of the total number of scanned pregnant women, 12.7% were found to have a report of abnormal U/S scans. Almost all of these cases (98.4%) were referred to a nearby hospital for confirmation of the diagnosis and subsequent management. This finding was by far lower than the 31.7% reported by a study in Philippines [13]. This huge difference can be explained by the fact that the Philippines study was conducted on a small sample size (460 pregnant women) while this Ethiopian study was conducted on larger sample size (12,975 pregnant women). Additionally, in the study conducted in the Philippines, specific and selected abnormalities were expected to be reported during 20–24th weeks of gestational ages, while this study was conducted during all gestational ages. Hence, malpresentations were not expected to be diagnosed during earlier gestational ages using U/S scans which might have lowered the proportion of reported abnormal findings [13].

The major abnormal U/S diagnoses responsible for referred pregnant women to nearby hospitals with functional EmONC services were malpresentations (8.9%) followed by multiple pregnancies in 1.3% of the cases. This finding is slightly different from a study in Guatemala which reported a 14.87% finding of non-cephalic presentations and a 0.54% rate of twins. This difference may be explained by the fact that all non-cephalic presentations were diagnosed and reported in the Guatemalan study, but only breech and transverse lie presentations were diagnosed and reported in this Ethiopian study. Similarly, the Guatemalan study reported only twin pregnancies while this Ethiopian study reported on all multifetal gestations [12].

The introduction of U/S scanning services at a semi-urban health center could have a contribution of preventing 1970 maternal morbidities and mortalities per 100,000 live births. This finding was much lower than the estimated maternal mortality prevention rate of 6300 per 100,000 live births in the Philippines [13]. This difference might be explained by the fact that the target population of both studies differ in terms of gestational weeks assessed [13]. Another study reported a reduction of maternal mortality rates from a national estimate of 110.86 per 100,000 live births to 97.98 to 102.77 per 100,000 live births, with the introduction of portable U/S equipment and the assignment of trained nurses in Guatemala [18].

The major possible causes of neonatal morbidity and mortality identified with U/S scans were fetal malpresentation (9.0%), followed by multiple pregnancies in 1.3% of cases. This finding is in alignment with the Philippines study which reported fetal malpresentations as the major abnormal U/S finding reported [13]. The introduction of U/S scanning services at semi-urban health centers has contributed to the possible prevention of 19.05 neonatal morbidities and mortalities per 1000 live births. This finding is lower than a study finding in the Philippines which reported 146 neonatal deaths averted per 1000 live births. This difference might be due to differences in sample size and gestational age of those assessed in the studies [13]. Another study in Guatemala reported a reduction in neonatal mortality after the introduction of portable U/S equipment following training of nurses [18].

### Strengths and limitations

The strengths of this retrospective facility-based study are in its description of rural residing pregnant women and their fetuses that have benefited from limited obstetric U/S services introduced when compared with their counterparts in semi-urban areas. In addition, the study used a large size of data and highlighted the contribution of prenatal U/S services in preventing maternal and neonatal catastrophic health outcomes during the immediate postnatal period within a low-income country. However, the study also has some known limitations. The first limitation is related to the study design which unlike randomized controlled trials, makes it difficult to claim causality. Since, the investigators decided to estimate averted catastrophic health outcomes using abnormal U/S scans applying criteria for the worst scenarios, this might cause an inflation of estimates. Additionally, the averted maternal and neonatal morbidities and mortalities might have been positively influenced by other interventions.

## Conclusions

This study describes prenatal U/S service beneficiaries after the introduction of the services in rural and semi-urban health centers in Ethiopia. The study estimates the averted catastrophic health outcomes which can potentially happen during the immediate post-partum period. After availing the appropriate U/S machines with essential supplies and capacitating mid-level healthcare providers (mainly midwives) on operating the machines, a significant number of high-risk pregnant women were identified on time and were treated or referred to health facilities with safe delivery services. This in turn may contribute to the prevention of maternal and neonatal morbidities and mortalities. Therefore, scaling-up limited obstetric U/S services in similar setups will contribute to the achievement of the Sustainable Development Goal (SDG) of reducing maternal mortality ratio and neonatal mortality rate by 2030. In addition, it is recommended community awareness be enhanced and pregnant women’s U/S service utilization be increased before the 24th week of gestation.

## Supplementary Information

**Additional file 1.** Ultrasound scan logbook

## Data Availability

All relevant data are within the paper. If more information on the datasets generated during and analyzed during the current study are required, they are available from the corresponding author on reasonable request.

## Abbreviations

ANC: Antenatal Care
EmONC: Emergency Obstetric and Newborn Care
IUFD: Intrauterine Fetal Death
LMP: Last Menstrual Period
OSCE: Objective Structured Clinical Examination
SNNP: Southern, Nations, Nationalities, and Peoples
SD: Standard Deviation
SDGs: Sustainable Development Goals
SSA: Sub-Sharan Africa
U/S: Ultrasound
USAID: United States Agency for International Development
WHO: World Health Organization
