# Supplementary material for: Contribution of portable obstetric ultrasound service innovation in averting maternal and neonatal morbidities and mortalities at semi-urban health centers of Ethiopia: a retrospective facility-based study
Source: BMC Pregnancy Childbirth. 2022 Apr 28;22:368. doi: 10.1186/s12884-022-04703-1 (PMC9052561; doi:10.1186/s12884-022-04703-1)
Supplement: Supplementary file 1 — Additional file 1. Ultrasound scan logbook [file 12884_2022_4703_MOESM1_ESM.pdf]

ULTRASOUND SCAN LOG

REGION______________________________________ **ZONE**____ **WOREDA**____

S.N	Date	Medical Record No. (MRN)	Patient Telephone No.	Patient Knows LMP?	GA given by LMP	GA Estimated on Ultrasound	GA by CRL	GA by BPD	GA by HC	GA by AC	GA by FL	Is this her first scan in this pregnancy?
				☐ Yes<br>☐ No								☐ Yes<br>☐ No<br>Write number of prior scans:________________________
				☐ Yes<br>☐ No								☐ Yes<br>☐ No<br>Write number of prior scans:________________________
				☐ Yes<br>☐ No								☐ Yes<br>☐ No<br>Write number of prior scans:________________________
				☐ Yes<br>☐ No								☐ Yes<br>☐ No<br>Write number of prior scans:________________________
				☐ Yes<br>☐ No								☐ Yes<br>☐ No<br>Write number of prior scans:________________________
				☐ Yes<br>☐ No								☐ Yes<br>☐ No<br>Write number of prior scans:________________________
				☐ Yes<br>☐ No								☐ Yes<br>☐ No<br>Write number of prior scans:________________________
				☐ Yes<br>☐ No								☐ Yes<br>☐ No<br>Write number of prior scans:________________________

ULTRASOUND SCAN LOG

HEALTH FACILITY____ INSTRUCTION: TICK (✓) ALL THAT IS APPLICABE

Her Current Trimester	Indication for Scanning	Ultrasound Diagnosis	Action	Reason for Referral	Outcomes
☐ 1st Trimester<br>☐ 2nd Trimester<br>☐ 3rd Trimester<br>☐ Pelvic Scan<br>☐ Others___________	☐ Routine<br>☐ High Risk pregnancy<br>☐ Trauma /Emergency<br>☐ Other:__________________	☐ Normal<br>☐ Breech<br>☐ Low-lying<br>☐ previa<br>☐ Multiple gestation<br>☐ Small for GA<br>☐ Abnormal fluid<br>☐ IUFD/Fetal demise<br>☐ Gross anomaly<br>☐ Risk of abortion<br>☐ Large for GA<br>☐ Pelvic Pathology<br>☐ Others: ________________	☐ Referred<br>☐ Managed in facility<br>☐ Follow-up<br>☐ No Action<br>☐ Appointment given (date)____________<br>☐ Follow-up call	☐ Unsure of findings<br>☐ Due to Detected diagnosis<br>☐ Facility limitation to handle<br>☐ Patient Preference<br>☐ Other reasons__________________	☐ Normal Vaginal Delivery<br>☐ Caesarean section<br>☐ Preterm/small baby<br>☐ MVA<br>☐ Fresh/Macerated still born<br>☐ Confirm Diagnosis<br>☐ Higher referral<br>☐ Other________________
☐ 1st Trimester<br>☐ 2nd Trimester<br>☐ 3rd Trimester<br>☐ Pelvic Scan<br>☐ Others___________	☐ Routine<br>☐ High Risk pregnancy<br>☐ Trauma /Emergency<br>☐ Other:__________________	☐ Normal<br>☐ Breech<br>☐ Low-lying<br>☐ previa<br>☐ Multiple gestation<br>☐ Small for GA<br>☐ Abnormal fluid<br>☐ IUFD/Fetal demise<br>☐ Gross anomaly<br>☐ Risk of abortion<br>☐ Large for GA<br>☐ Pelvic Pathology<br>☐ Others: ________________	☐ Referred<br>☐ Managed in facility<br>☐ Follow-up<br>☐ No Action<br>☐ Appointment given (date)____________<br>☐ Follow-up call	☐ Unsure of findings<br>☐ Due to Detected diagnosis<br>☐ Facility limitation to handle<br>☐ Patient Preference<br>☐ Other reasons__________________	☐ Normal Vaginal Delivery<br>☐ Caesarean section<br>☐ Preterm/small baby<br>☐ MVA<br>☐ Fresh/Macerated still born<br>☐ Confirm Diagnosis<br>☐ Higher referral<br>☐ Other________________
☐ 1st Trimester<br>☐ 2nd Trimester<br>☐ 3rd Trimester<br>☐ Pelvic Scan<br>☐ Others___________	☐ Routine<br>☐ High Risk pregnancy<br>☐ Trauma /Emergency<br>☐ Other:__________________	☐ Normal<br>☐ Breech<br>☐ Low-lying<br>☐ previa<br>☐ Multiple gestation<br>☐ Small for GA<br>☐ Abnormal fluid<br>☐ IUFD/Fetal demise<br>☐ Gross anomaly<br>☐ Risk of abortion<br>☐ Large for GA<br>☐ Pelvic Pathology<br>☐ Others: ________________	☐ Referred<br>☐ Managed in facility<br>☐ Follow-up<br>☐ No Action<br>☐ Appointment given (date)____________<br>☐ Follow-up call	☐ Unsure of findings<br>☐ Due to Detected diagnosis<br>☐ Facility limitation to handle<br>☐ Patient Preference<br>☐ Other reasons__________________	☐ Normal Vaginal Delivery<br>☐ Caesarean section<br>☐ Preterm/small baby<br>☐ MVA<br>☐ Fresh/Macerated still born<br>☐ Confirm Diagnosis<br>☐ Higher referral<br>☐ Other________________
☐ 1st Trimester<br>☐ 2nd Trimester<br>☐ 3rd Trimester<br>☐ Pelvic Scan<br>☐ Others___________	☐ Routine<br>☐ High Risk pregnancy<br>☐ Trauma /Emergency<br>☐ Other:__________________	☐ Normal<br>☐ Breech<br>☐ Low-lying<br>☐ previa<br>☐ Multiple gestation<br>☐ Small for GA<br>☐ Abnormal fluid<br>☐ IUFD/Fetal demise<br>☐ Gross anomaly<br>☐ Risk of abortion<br>☐ Large for GA<br>☐ Pelvic Pathology<br>☐ Others: ________________	☐ Referred<br>☐ Managed in facility<br>☐ Follow-up<br>☐ No Action<br>☐ Appointment given (date)____________<br>☐ Follow-up call	☐ Unsure of findings<br>☐ Due to Detected diagnosis<br>☐ Facility limitation to handle<br>☐ Patient Preference<br>☐ Other reasons__________________	☐ Normal Vaginal Delivery<br>☐ Caesarean section<br>☐ Preterm/small baby<br>☐ MVA<br>☐ Fresh/Macerated still born<br>☐ Confirm Diagnosis<br>☐ Higher referral<br>☐ Other________________
☐ 1st Trimester<br>☐ 2nd Trimester<br>☐ 3rd Trimester<br>☐ Pelvic Scan<br>☐ Others___________	☐ Routine<br>☐ High Risk pregnancy<br>☐ Trauma /Emergency<br>☐ Other:__________________	☐ Normal<br>☐ Breech<br>☐ Low-lying<br>☐ previa<br>☐ Multiple gestation<br>☐ Small for GA<br>☐ Abnormal fluid<br>☐ IUFD/Fetal demise<br>☐ Gross anomaly<br>☐ Risk of abortion<br>☐ Large for GA<br>☐ Pelvic Pathology<br>☐ Others: ________________	☐ Referred<br>☐ Managed in facility<br>☐ Follow-up<br>☐ No Action<br>☐ Appointment given (date)____________<br>☐ Follow-up call	☐ Unsure of findings<br>☐ Due to Detected diagnosis<br>☐ Facility limitation to handle<br>☐ Patient Preference<br>☐ Other reasons__________________	☐ Normal Vaginal Delivery<br>☐ Caesarean section<br>☐ Preterm/small baby<br>☐ MVA<br>☐ Fresh/Macerated still born<br>☐ Confirm Diagnosis<br>☐ Higher referral<br>☐ Other________________
☐ 1st Trimester<br>☐ 2nd Trimester<br>☐ 3rd Trimester<br>☐ Pelvic Scan<br>☐ Others___________	☐ Routine<br>☐ High Risk pregnancy<br>☐ Trauma /Emergency<br>☐ Other:__________________	☐ Normal<br>☐ Breech<br>☐ Low-lying<br>☐ previa<br>☐ Multiple gestation<br>☐ Small for GA<br>☐ Abnormal fluid<br>☐ IUFD/Fetal demise<br>☐ Gross anomaly<br>☐ Risk of abortion<br>☐ Large for GA<br>☐ Pelvic Pathology<br>☐ Others: ________________	☐ Referred<br>☐ Managed in facility<br>☐ Follow-up<br>☐ No Action<br>☐ Appointment given (date)____________<br>☐ Follow-up call	☐ Unsure of findings<br>☐ Due to Detected diagnosis<br>☐ Facility limitation to handle<br>☐ Patient Preference<br>☐ Other reasons__________________	☐ Normal Vaginal Delivery<br>☐ Caesarean section<br>☐ Preterm/small baby<br>☐ MVA<br>☐ Fresh/Macerated still born<br>☐ Confirm Diagnosis<br>☐ Higher referral<br>☐ Other________________
☐ 1st Trimester<br>☐ 2nd Trimester<br>☐ 3rd Trimester<br>☐ Pelvic Scan<br>☐ Others___________	☐ Routine<br>☐ High Risk pregnancy<br>☐ Trauma /Emergency<br>☐ Other:__________________	☐ Normal<br>☐ Breech<br>☐ Low-lying<br>☐ previa<br>☐ Multiple gestation<br>☐ Small for GA<br>☐ Abnormal fluid<br>☐ IUFD/Fetal demise<br>☐ Gross anomaly<br>☐ Risk of abortion<br>☐ Large for GA<br>☐ Pelvic Pathology<br>☐ Others: ________________	☐ Referred<br>☐ Managed in facility<br>☐ Follow-up<br>☐ No Action<br>☐ Appointment given (date)____________<br>☐ Follow-up call	☐ Unsure of findings<br>☐ Due to Detected diagnosis<br>☐ Facility limitation to handle<br>☐ Patient Preference<br>☐ Other reasons__________________	☐ Normal Vaginal Delivery<br>☐ Caesarean section<br>☐ Preterm/small baby<br>☐ MVA<br>☐ Fresh/Macerated still born<br>☐ Confirm Diagnosis<br>☐ Higher referral<br>☐ Other________________
☐ 1st Trimester<br>☐ 2nd Trimester<br>☐ 3rd Trimester<br>☐ Pelvic Scan<br>☐ Others___________	☐ Routine<br>☐ High Risk pregnancy<br>☐ Trauma /Emergency<br>☐ Other:__________________	☐ Normal<br>☐ Breech<br>☐ Low-lying<br>☐ previa<br>☐ Multiple gestation<br>☐ Small for GA<br>☐ Abnormal fluid<br>☐ IUFD/Fetal demise<br>☐ Gross anomaly<br>☐ Risk of abortion<br>☐ Large for GA<br>☐ Pelvic Pathology<br>☐ Others: ________________	☐ Referred<br>☐ Managed in facility<br>☐ Follow-up<br>☐ No Action<br>☐ Appointment given (date)____________<br>☐ Follow-up call	☐ Unsure of findings<br>☐ Due to Detected diagnosis<br>☐ Facility limitation to handle<br>☐ Patient Preference<br>☐ Other reasons__________________	☐ Normal Vaginal Delivery<br>☐ Caesarean section<br>☐ Preterm/small baby<br>☐ MVA<br>☐ Fresh/Macerated still born<br>☐ Confirm Diagnosis<br>☐ Higher referral<br>☐ Other________________
☐ 1st Trimester<br>☐ 2nd Trimester<br>☐ 3rd Trimester<br>☐ Pelvic Scan<br>☐ Others___________	☐ Routine<br>☐ High Risk pregnancy<br>☐ Trauma /Emergency<br>☐ Other:__________________	☐ Normal<br>☐ Breech<br>☐ Low-lying<br>☐ previa<br>☐ Multiple gestation<br>☐ Small for GA<br>☐ Abnormal fluid<br>☐ IUFD/Fetal demise<br>☐ Gross anomaly<br>☐ Risk of abortion<br>☐ Large for GA<br>☐ Pelvic Pathology<br>☐ Others: ________________	☐ Referred<br>☐ Managed in facility<br>☐ Follow-up<br>☐ No Action<br>☐ Appointment given (date)____________<br>☐ Follow-up call	☐ Unsure of findings<br>☐ Due to Detected diagnosis<br>☐ Facility limitation to handle<br>☐ Patient Preference<br>☐ Other reasons__________________	☐ Normal Vaginal Delivery<br>☐ Caesarean section<br>☐ Preterm/small baby<br>☐ MVA<br>☐ Fresh/Macerated still born<br>☐ Confirm Diagnosis<br>☐ Higher referral<br>☐ Other________________
